# The Basic/Helix-Loop-Helix Protein Family in *Gossypium*: Reference Genes and Their Evolution during Tetraploidization

**DOI:** 10.1371/journal.pone.0126558

**Published:** 2015-05-18

**Authors:** Qian Yan, Hou-Sheng Liu, Dan Yao, Xin Li, Han Chen, Yang Dou, Yi Wang, Yan Pei, Yue-Hua Xiao

**Affiliations:** Biotechnology Research Center, Chongqing Key Laboratory of Application and Safety Control of Genetically Modified Crops, Southwest University, Beibei, Chongqing, China; Zhejiang A & F University, CHINA

## Abstract

Basic/helix-loop-helix (bHLH) proteins comprise one of the largest transcription factor families and play important roles in diverse cellular and molecular processes. Comprehensive analyses of the composition and evolution of the bHLH family in cotton are essential to elucidate their functions and the molecular basis of cotton development. By searching bHLH homologous genes in sequenced diploid cotton genomes (*Gossypium raimondii* and *G*. *arboreum*), a set of cotton bHLH reference genes containing 289 paralogs were identified and named as *GobHLH001-289*. Based on their phylogenetic relationships, these cotton bHLH proteins were clustered into 27 subfamilies. Compared to those in *Arabidopsis* and cacao, cotton bHLH proteins generally increased in number, but unevenly in different subfamilies. To further uncover evolutionary changes of bHLH genes during tetraploidization of cotton, all genes of S5a and S5b subfamilies in upland cotton and its diploid progenitors were cloned and compared, and their transcript profiles were determined in upland cotton. A total of 10 genes of S5a and S5b subfamilies (doubled from A- and D-genome progenitors) maintained in tetraploid cottons. The major sequence changes in upland cotton included a 15-bp in-frame deletion in *GhbHLH130D* and a long terminal repeat retrotransposon inserted in *GhbHLH062A*, which eliminated *GhbHLH062A* expression in various tissues. The S5a and S5b bHLH genes of A and D genomes (except *GobHLH062*) showed similar transcription patterns in various tissues including roots, stems, leaves, petals, ovules, and fibers, while the A- and D-genome genes of *GobHLH110* and *GobHLH130* displayed clearly different transcript profiles during fiber development. In total, this study represented a genome-wide analysis of cotton bHLH family, and revealed significant changes in sequence and expression of these genes in tetraploid cottons, which paved the way for further functional analyses of bHLH genes in the cotton genus.

## Introduction

Basic/helix-loop-helix (bHLH) transcription factors, named from their signature bHLH domains, are ubiquitously distributed in major eukaryotes and involved in diverse cellular and molecular processes [1–3]. A bHLH domain generally comprises around 60 amino acids and two functionally distinct segments, i.e., the basic and helix-loop-helix regions. Structural analyses have indicated that the basic region forms the major interface contacting DNA, whereas the helix-loop-helix region mediates protein-protein interactions regulating DNA binding activity [3]. By interacting with DNA and different proteins simultaneously, bHLH proteins frequently act as central integrators in gene regulation networks [1,2,4–7]. For example, phytochrome-interacting factors (PIFs), the major regulators of plant photomorphogenic development, interact with multiple regulatory proteins (such as DELLA, HY5, phy, BZR1) from different pathways and integrate diverse signals to control plant growth [1].

With more and more genomes sequenced, increasing number of bHLH proteins have been identified and employed in classification and evolutionary comparison across a wide range of organisms [3,8–16]. Compared to fungi and metazoans, the bHLH family expands significantly in higher plants, harboring 88 to 289 bHLH genes in a single genome [9,12,13]. Based on their evolutionary relationships, bHLH domains identified from representative species (*Arabidopsis*, poplar, rice, moss and algae) were classified into 32 subfamilies with 2 moss-specific (S6 and S29), and 1 algae-specific subfamily (S32) [13].

Cotton is the leading fiber crop and provides the majority of natural fibers in the worldwide textile market. Among around 50 species in cotton (*Gossypium*) genus, two diploids (*G*. *arboreum* and *G*. *herbaceum*, 2n = 2X = AA = 26) and two allotetraploids (upland cotton, *G*. *hirsutum*, and sea island cotton, *G*. *barbadense*, 2n = 4X = AADD = 52), have been cultivated to produce economically valuable fibers [17]. It is believed that allotetraploid cottons all derive from an interspecific hybrid formed during the Pleistocene (1–2 millions of years ago). *G*. *raimondii* is the closest living relative of the D-genome donor of allotetraploid cottons, but it does not produce significantly elongated fibers as the A-genome donors (*G*. *arboreum* and *G*. *herbaceum*) [17]. Upland cotton is most widely used in the modern cotton industry, and accounts for most of the world’s cotton yield. Compared to their diploid progenitors, upland cotton and sea island cotton have significantly higher yield and fiber quality. Agronomists and biologists have long been interested in genomic evolution of tetraploidization and domestication of cottons [17–22]. Transcriptomic analyses indicated that several pathways were up-regulated in developing fibers and homoeologous genes might be differentially regulated in tetraploid cotton tissues, implying that genes from different genomes may act synergistically to enhance fiber production in tetraploid cottons [19–26]. However, the importance of these up-regulated pathways and the mechanisms to control these pathways still need to be elucidated. As one of the largest transcription factor families in plants, bHLH proteins may play important roles in regulating various pathways and cotton development. Comprehensive analysis of cotton bHLH proteins and their evolutionary changes during tetraploidization may help to reveal molecular mechanisms underlying the varied pathways and super quality and yield in modern tetraploid cottons. On the other hand, evolutionary effect of alloploidy has long been an attracting theme in plant biology [18,27–32], since an estimate of 30–70% plant species, including many important crops such as cotton, wheat, oilseed rape, and tobacco, are of alloploid origin. Analyses of genetic and transcriptional alterations of bHLH homoeologous genes in allotetraploid cotton may provide useful clues to elucidate the influence of alloploidy in plant evolution and the molecular basis of speciation and domestication of alloploid crops.

In this study, we identified bHLH genes comprehensively from the known cotton sequences, including the genomes of *G*. *raimondii* and *G*. *arboreum* which were recently sequenced [18,33,34]. A set of *Gossypium* bHLH reference genes were constructed and employed to analyze their evolutionary relationships with homologs from the model plant *Arabidopsis*, and cacao (*Theobroma cacao*), a sequenced species most closely related to the cotton genus. To explore evolutionary changes of bHLH genes during tetraploidization, all genes of two subfamilies (S5a and S5b) in upland cotton and its diploid progenitors were cloned and compared, and transcription profiles of the genome-specific orthologous genes were further determined in upland cotton.

## Materials and Methods

### Sequence sources

All sequence data were obtained from the internet (S1 Table). *Arabidopsis* bHLH proteins and reference bHLH sequences of *Oryza sativa*, *Physcomitrella patens*, and *Chlamydomonas reinhardtii* (S2 Table) were retrieved from Phytozome (http://www.phytozome.net/search.php) [35] according to Carretero-Paulet et al [13]. The annotated genome sequences of *T*. *cacao* and *G*. *raimondii* (Gr-JGI) were downloaded from Phytozome [18,35,36]. The annotations of *G*. *raimondii* and *G*. *arboreum* genomes (Gr-CGP and Ga-CGP) were from the Cotton Genome Project in the Institute of Cotton Research of Chinese Academy of Agricultural Sciences (http://cgp.genomics.org.cn/page/species/download.jsp?category=raimondii and = arboreum, respectively) [33,34]. Upland cotton unigenes (Gh-Uni) were obtained from Plant Transcription Factor Database (PlantTFDB, http://planttfdb.cbi.pku.edu.cn/family.php?sp=Ghi&fam=bHLH) [37]. *Gossypium* bHLH contigs (Go-con) and mRNA sequences were retrieved from Cottongen (http://www.cottongen.org/retrieve/sequences) by searching sequences containing bHLH domain (IPR011598) [38].

### Identification of bHLH proteins and corresponding bHLH domains

According to classification of plant bHLH proteins reported previously [13], 32 representative bHLH domains (one per subfamily) and three *Arabidopsis* orphans were selected to constitute a set of probe sequences (S2 Table). To identify bHLH proteins from annotated genomes (*G*. *raimondii*, *G*. *arboreum* and *T*. *cacao*), all probe bHLHs were employed to query primary-transcript-only proteins of the sequenced genomes by a standalone BLAST program [39]. For each genome, repeated entries were eliminated by Microsoft Excel program, and putative bHLH domains were retrieved according to two-sequence alignments in BLAST. The resulting bHLH sequences were further aligned with all probe sequences and *Arabidopsis* bHLH domains. The sequences conforming to the following rules were validated as bHLH domains. A bHLH domain should contain 1) at least two continuous sub-regions of basic, helix1, and helix2 and 2) over 60% consensus amino acid residuals identified in plant bHLHs by Carretero-Paulet et al [13].

To determine *Gossypium* reference bHLH genes, cotton bHLH proteins identified from various sources (Gr-JGI, Gr-CGP, Ga-CGP, Gh-Uni, Go-con, and mRNA) were aligned using AlignX program in Vector II software (Invitrogen). The branch lengths (BL) in the alignment guide tree reflecting the genetic divergences between sequences were employed as an arbitrary standard to group corresponding sequences. The proteins with BL<0.03, 0.03 to 0.15, and >0.15 were regarded as originating from an allele gene, orthologous genes of different genomes and different paralogous genes, respectively. For each orthologous group, a single representative member (mainly from Gr-JGI) was selected as reference gene, and the *Gossypium* reference bHLH genes included all non-overlapping paralogous genes (S3 Table).

### Phylogenetic analysis and classification of *Gossypium* bHLHs

Phylogenetic analysis was performed using MEGA6.0 [40]. All cotton reference bHLH domains were aligned with the bHLH domains from *Arabidopsis* and *T*. *cacao*. Since members of several plant bHLH subfamilies (S6, S8, S29 and S32) were not identified in *Arabidopsis* [13], each two representative sequences of these subfamilies from other plants (S4 Table) were also included in the multiple sequence alignment. The alignment was performed using clustalW with default settings. Phylogenetic trees were constructed and tested by neighbor joining (NJ), maximum parsimony (MP), and maximum likelihood (ML) methods, and bootstrap test was set as 1000 replicates. Classification of bHLH proteins was performed according to evolutionary relationships of bHLH domains. The bHLHs on a branch supported by at least two methods and with high bootstrap (>88%) in NJ test were clustered into a subfamily. The resultant bHLH subfamilies were compared to those determined by Carretero-Paulet et al [13], and named accordingly.

### Cloning and sequence analysis of *Gossypium* S5a and S5b bHLH genes

Cotton DNAs were extracted from fresh leaves using a plant DNA extraction kit (Aidlab, Beijing, China). The S5a and S5b bHLH genes (except for *GhbHLH062A*) from *G*. *hirsutum*, *G*. *arboreum*, and *G*. *raimondii* were amplified with primers encompassing the coding regions (S5 Table). The 25-μl PCR reactions included 100 ng cotton genomic DNA, 1×PrimerSTAR mix (TaKaRa), 200 nM upstream and downstream primers. The PCR thermocycling parameters were as follows: 98°C for 1 min, followed by 35 cycles of 98°C for 10s, 55°C for 15 s and 72°C for 1 min, and a final extension of 3 min at 72°C. After A-tailing, all PCR products were cloned into pGEM-T (Promega) and sequenced by Invitrogen (Shanghai, China).

For *GhbHLH062A*, we first cloned the 3’-end of coding region using the A-specific primer and downstream coding-region primer. The upstream sequences were then amplified by two rounds of Y-shaped adaptor dependent extension (YADE) [41] until a long terminal repeat (LTR) retrotransposon insertion was found. Finally, the 5’-end of coding region were amplified using the upstream coding-region primer and a LTR primer.

The exon sequences and ORFs were determined by comparing genomic sequences to EST, mRNA or homologous proteins using BLAST program in NCBI. The deduced protein sequences were aligned and subjected to construction and test of phylogenetic tree using NJ method in MEGA6.0 [40]. The genome origin (A or D) of a certain gene from tetraploid cotton was determined according to its evolutionary relatedness to the orthologous genes from progenitor diploids (*G*. *arboreum* and *G*. *raimondii*).

To detect a certain gene in tetraploid and diploid cottons, genome-specific primers were designed to amplify fragments of 100 to 250 bp from genomic DNAs using 2×Taq PCR mixture (Tiangen, China). The PCRs contained around 100 ng genomic DNAs and 200 nM upstream and downstream primers, and amplified for 30 cycles of 94°C for 30 s, 56°C for 30 s, and 72°C for 30 s. The PCR products were detected by EtBr staining in agarose gel.

Sequences of all PCR primers employed in this study are shown in S5 Table.

### RNA extraction and real-time RT-PCR analysis

Upland cotton RNAs were extracted from roots, stems, leaves, petals, ovules, and fibers of different developmental stages using a rapid plant RNA extraction kit (Aidlab, Beijing, China). The cDNAs were synthesized from total RNA using a first-strand cDNA synthesis kit (TaKaRa, Dalian, China), and then subjected to real-time PCR analyses. Real-time PCRs were performed on a CFX96 real-time PCR detection system using SYBR Green Supermix (Bio-Rad, CA, USA) according to the manufacturer’s introductions. The thermocycling parameters were as follows: 95°C for 2 min, followed by 40 cycles of 95°C for 10 s, 57°C for 20 s, followed by a standard melting curve to monitor PCR specificity. Cotton histone3 (AF024716) [42] and GhUBQ14 [43] genes were amplified as internal standards. Reactions were performed for three replicates. Data were analyzed using the software Bio-Rad CFX Manager 2.0 provided by the manufacturer.

## Results

### Identification of cotton bHLH genes in *Gossypium* genomes

The D and A genomes of diploid cottons were recently sequenced by different projects (S1 Table) [18,33,34]. To perform a comprehensive analysis of cotton bHLH family, a set of plant bHLH probe sequences were employed to identify bHLH genes from annotated genomes of *G*. *raimondii* and *G*. *arboreum*. Consequently, 272, 255, and 256 distinct bHLH proteins were identified in the genomic sequences from Gr-JGI, Gr-CGP, and Ga-CGP, respectively. These sequences were further aligned with bHLH proteins annotated in *Gossypium* EST contigs (Go-con), *G*. *hirsutum* unigenes (Gh-Uni), and cotton mRNAs. Finally, 919 *Gossypium* bHLH proteins were clustered into 289 orthologous groups (S3 Table). Each orthologous group in the alignment might represent a distinct bHLH gene in *Gossypium* reference genome. Therefore, we selected a single representative from each orthologous group to constitute *Gossypium* reference bHLH genes, which were coded alphabetically as *GobHLH001-289* (S3 Table).

As shown in Table 1 and S3 Table, most bHLH genes (>89%) had orthologous or overlapped genes from other source(s). All proteins from Go-con, Gh-Uni, and mRNA could be assigned to a certain gene in sequenced genomes, suggesting that most bHLH-coding genes in *Gossypium* might have been revealed by genome sequencing. On the other hand, none of the three genomic sequencing projects had annotated all the bHLH reference genes, indicating that the cotton genome sequences were still relatively fragmented and might have missed a portion of information.

**Table 1:** Numbers and overlapping of cotton bHLH genes from different sources.

	Gr-JGI	Gr-CGP	Ga-CGP	Gh-Uni	Go-con	mRNA
Gr-JGI	272	249	243	79	28	4
Gr-CGP		255(257)*	236	78	27	4
Ga-CGP			256(259)*	79	28	4
Gh-Uni				79(92)	27	4
Go-con					28(29)	2
mRNA						4(10)

Numbers of non-overlapping paralogous genes from a certain source are shown on the diagonal line with the initial sequence number in brackets. Other numbers indicate overlapped or orthologous genes between two sources.

* The tandem repeat genes encoding identical proteins and orthologous to a single protein from other sources are regarded as a single gene.

### Phylogenetic analysis and classification of *Gossypium* bHLH family

**Fig 1:**
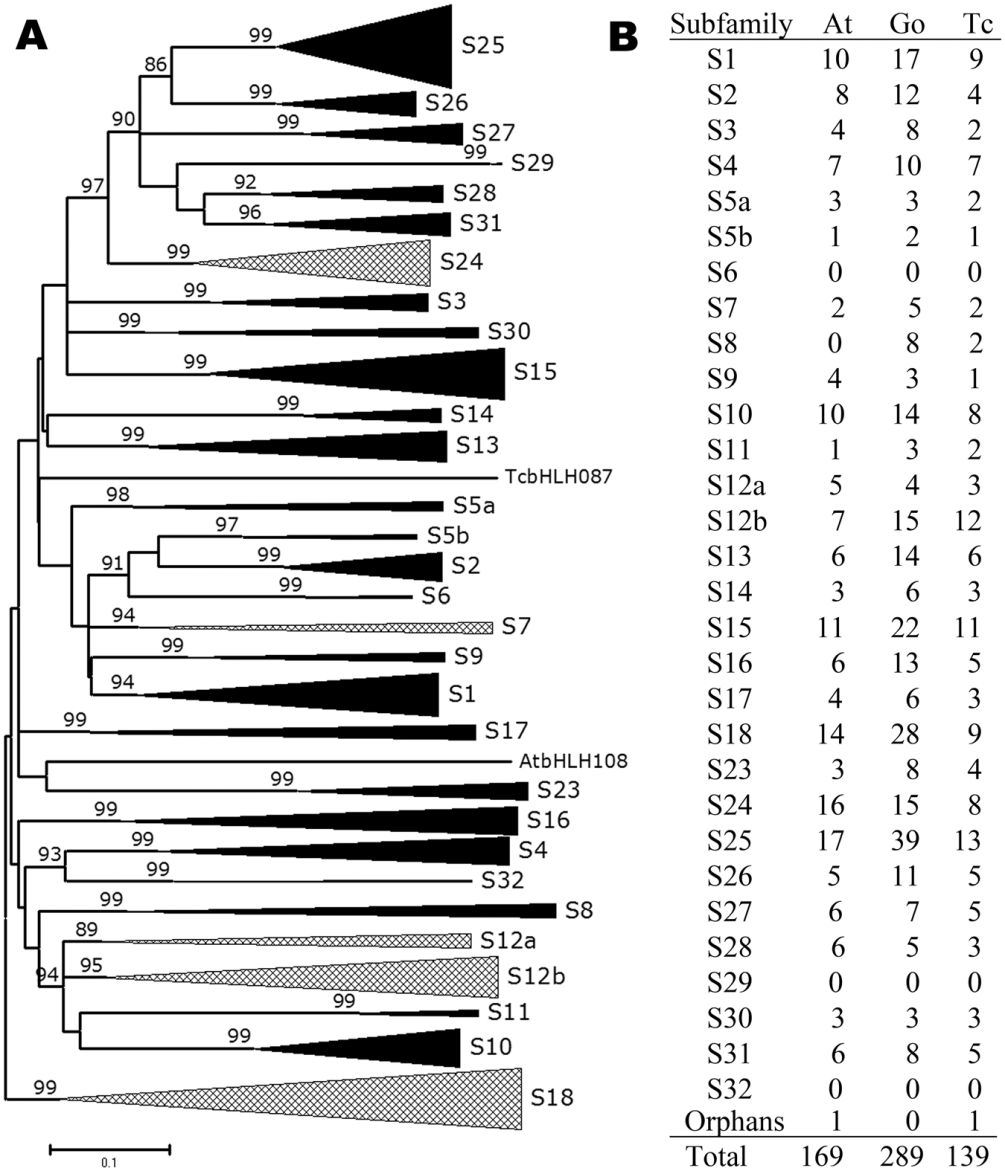
Phylogenetic relationships and classification of *Arabidopsis*, *Gossypium* and *Theobroma* bHLH domains. A, NJ tree of 605 bHLH domains (169 from *A*. *thaliana*, 289 from *Gossypium*, 139 from *T*. *cacao*, 4 from *P*. *patens*, 2 from *C*. *reinhardtii*, and 2 from *O*. *sativa*). Subfamilies are collapsed and represented as triangles with both depth and width proportional to sequence divergence and size, respectively. Orphans are represented as single lines. The scale bar indicates the estimated number of amino acid replacements per site. For subfamilies S6, S29 and S32, only 2 reference sequences from *P*. *patens* or *C*. *reinhardtii* are used in the alignment. The alignment used for tree construction and the full representation of the tree are shown in S1 and S2 Figs, respectively. B, Member numbers of different bHLH subfamilies in *A*. *thaliana* (At), *Gossypium* (Go) and *T*. *cacao* (Tc).

To analyze evolutionary relationship of *Gossypium* bHLH proteins, 289 *Gossypium* reference bHLH domains were aligned with bHLHs from *Arabidopsis* and cacao, and representative sequences of S6, S8, S29, and S32 bHLH subfamilies from *P*. *patens*, *C*. *reinhardtii*, and *O*. *sativa* (S1 Fig). Based on the resultant phylogenetic tree (Fig 1A; S2 Fig), 605 bHLH sequences were grouped into 30 subfamilies (Fig 1A and 1B; S6 Table). The majority of plant bHLH subfamilies determined by Carretero-Paulet et al [13] remained in our classification, except that S5 and S12 were divided into two subfamilies (S5a and S5b, S12a and S12b, respectively), and that S18, S19, S20, S21 and S22 were merged into a single subfamily S18.

In the phylogenetic tree, *Gossypium* bHLHs are assigned to 27 subfamilies along with those from *Theobroma* and *Arabidopsis* (Fig 1A; S2 Fig). Although *Gossypium* contains many more bHLH members, the subfamily numbers in *Gossypium* and *Theobroma* are the same (27 subfamilies), and only one (S8) more than that in *Arabidopsis* (S6 Table). Consistent with the recent genome polyploidization in *Gossypium* [18,33,34], *Gossypium* bHLH subfamily members generally increase in number, compared to *Theobroma*. However, this expansion (1- to 4-fold in gene number) is uneven, suggesting that the extent of gene deletion after polyploidization varies among subfamilies (Fig 1B).

### Origin and variations of S5a and S5b bHLH genes in tetraploid cottons

**Fig 2:**
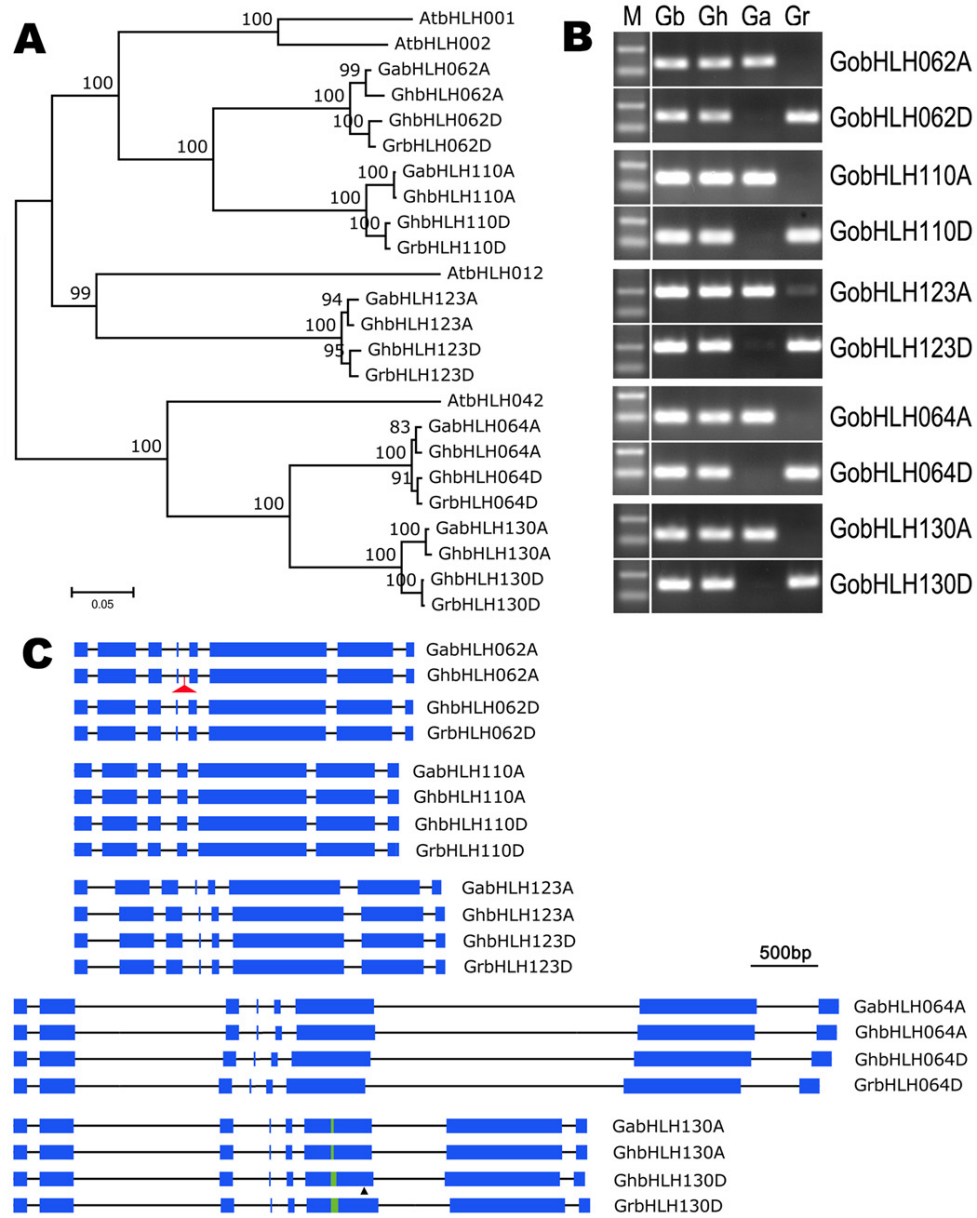
S5a and S5b bHLH genes in *Gossypium*. A, NJ tree of S5a and S5b bHLH proteins from *A*. *thaliana* and *Gossypium*. The scale bar indicates the estimated number of amino acid replacements per site. The tree was constructed and tested using the alignment shown in S3 Fig. Percentage of supported bootstrap for every branch in a test of 1000 replicates is indicated. B, PCR confirmation of the genome-specific S5a and S5b bHLH genes in tetraploid cottons *G*. *barbadense* (Gb) and *G*. *hirsutum* (Gh), and their diploid progenitors *G*. *arboreum* (Ga) and *G*. *raimondii* (Gr). M, DNA marker with two bands of 250 bp (upper) and 100 bp (lower) indicated. C, Gene structures of cotton S5a and S5b bHLH genes. The sequences corresponding to the ORFs are depicted proportionally. Exons and introns are represented by blue bars and black lines, respectively. Red triangle indicates the long terminal repeat (LTR) retrotransposon inserted in *GhbHLH062A* (Details are shown in S4 Fig). For *GobHLH130*s, the simple sequence repeats (GAA)_n_ of various lengths in the 6^th^ exon are presented in green and a black triangle directs the site of a 15-bp deletion in *GhbHLH130D* (see details in S5 Fig).

Cotton fibers are ovule epidermal trichomes and flavonoids may be involved in the regulation of fiber development [44,45]. Thus, we selected S5a and S5b bHLHs, acting as important regulators of trichome differentiation and flavonoid biosynthesis in *Arabidopsis* (S6 Table) [4,46,47], to explore evolutionary changes of bHLH genes in tetraploid cottons. All bHLH genes of S5a and S5b subfamilies (*GobHLH062*, *GobHLH064*, *GobHLH110*, *GobHLH123* and *GobHLH130*) were cloned from upland cotton and its diploid progenitors (*G*. *arboreum* and *G*. *raimondii*). These sequences were deposited in GenBank under the accession nos KP698854-KP698873. Consistent with the number of reference genes, both diploid cottons contained five genes, while upland cotton harbored 10 genes of S5a and S5b bHLH subfamilies. Based on deduced protein sequences, these cotton bHLH genes could be assigned to five orthologous groups, and each group included four genes, two from each genome (A or D; Fig 2A; S3 Fig). All of these genes could be also detected in another tetraploid species (*G*. *barbadense*) using genome-specific primers (Fig 2B). These data suggest that bHLH genes doubled by tetraploidization are generally maintained in tetraploid cottons. For the convenience of discrimination, we name a certain gene like *GhbHLH062A*, i.e., the abbreviation of species (the first two letters) plus reference gene code plus genome name (the last letter).

The structures of S5a and S5b bHLH genes from different cotton species are shown in Fig 2C. The exon-intron patterns of these genes are highly conserved, except that the 4^th^ intron is lost in *GobHLH110*s. Comparing to their orthologs from diploid cottons, there exist three major sequence variations, and several single-nucleotide changes in the upland cotton genes. Firstly, a LTR retrotransposon (~5 kb in length) is inserted in the 4^th^ intron of *G*. *hirsutum bHLH062A* (Fig 2C). As shown in S4 Fig, this LTR retrotransposon insertion exists in different *G*. *hirsutum* lines and also in *G*. *barbadense*, suggesting that this LTR retrotransposon duplication is a common evolutionary event during tetraploidization. Secondly, a 15-bp fragment is deleted in the 6^th^ exon of *GhbHLH130D* (Fig 2C). This deletion may be specifically in the *G*. *hirsutum* lineage, as it is detected in two *G*. *hirsutum* lines, but not in *G*. *barbadense* and *G*. *raimondii* (S5 Fig). Finally, the length of a simple sequence repeats (GAA)_n_ in the 6^th^ exon of *GhbHLH130D* is 12-bp shorter than that of *GrbHLH130D* (Fig 2C).

### Transcriptional profiling of S5a and S5b bHLH genes in upland cotton

**Fig 3:**
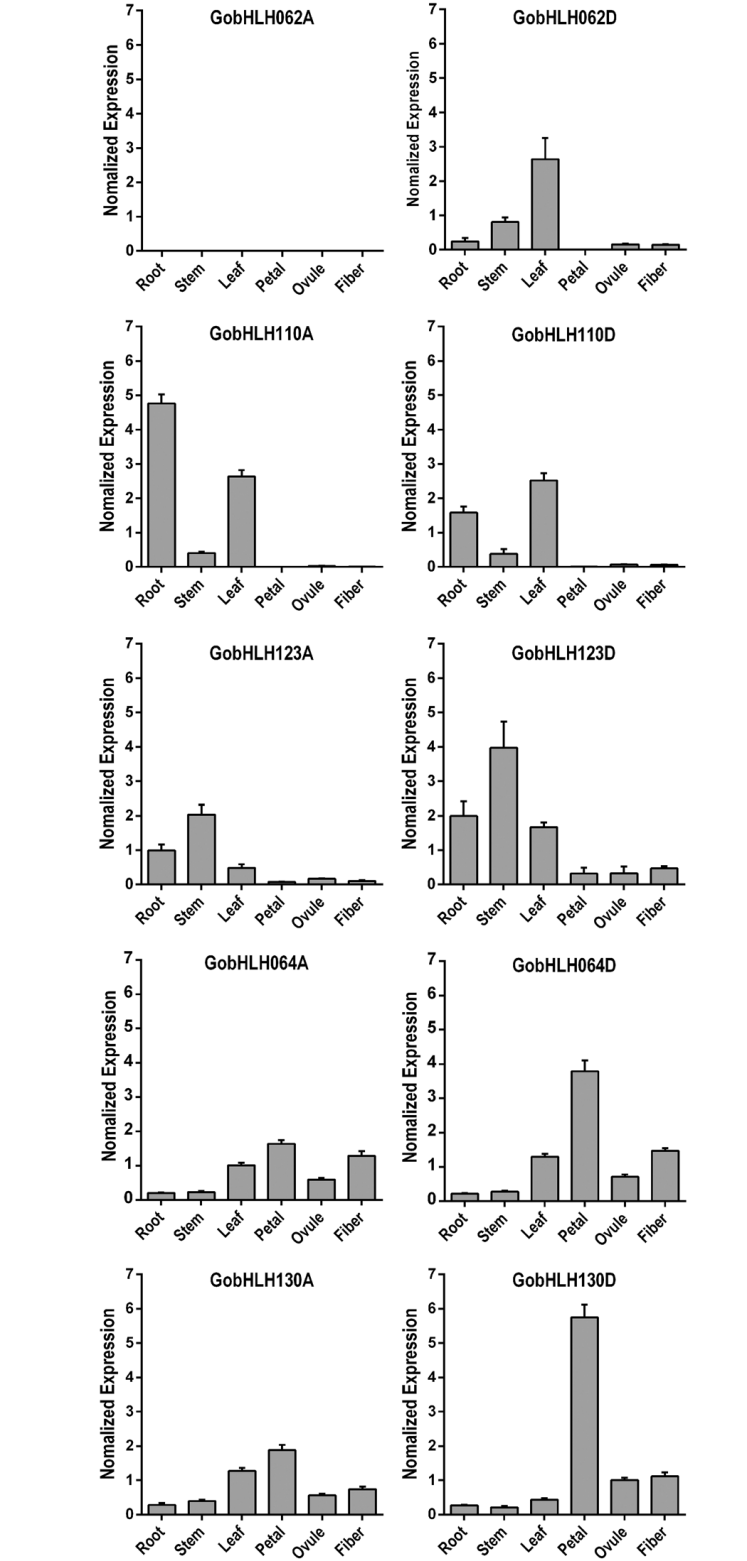
Expression profiles of S5a and S5b bHLH genes in various upland cotton tissues. Genome-specific primers were employed to detect relative transcript levels of 10 S5a and S5b bHLH genes in various tissues of upland cotton. Both cotton histone3 (AF024716) [42] and GhUBQ14 [43] genes were amplified and set as references. Error bars indicate SEM of three technical replicates.

**Fig 4:**
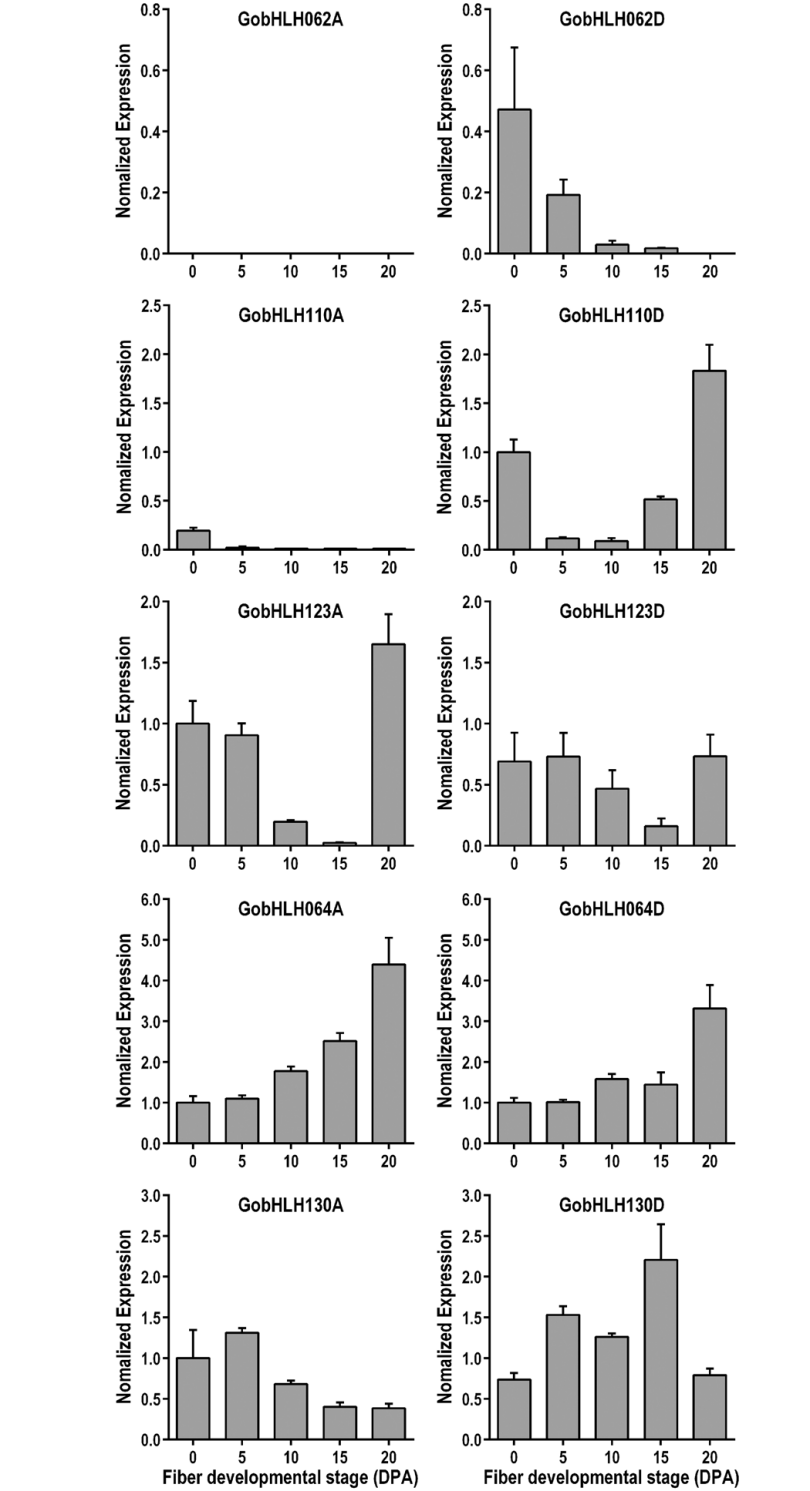
Expression profiles of S5a and S5b bHLH genes at different fiber developmental stages. Relative transcript levels of 10 S5a and S5b bHLH genes at different fiber developmental stages in upland cotton were detected by qRT-PCR. DPA, day(s) post anthesis. Total RNAs were extracted from ovules with fiber initials at anthesis (0 DPA) or fibers of different developmental stages (5~20 DPA). Data were analyzed and presented as in Fig 3.

To elucidate whether the doubled bHLH genes were differentially regulated at transcriptional level in tetraploid cotton, real-time RT-PCR was employed to detect the transcript levels of 10 S5a and S5b bHLH genes in various tissues and at different fiber developmental stages (Figs 3 and 4). The expression of *GhbHLH062A* was totally undetectable in all investigated tissues, suggesting that the LTR retrotransposon insertion in this gene might lead to gene disruption and loss of function. The rest nine bHLH genes all showed significant expression in certain tissues and the expression levels varied in a developmentally regulated pattern. Four pairs of homoeologous bHLH genes from different genomes (*GobHLH064A* and *D*, *GobHLH110A* and *D*, *GobHLH123A* and *D*, and *GobHLH130A* and *D*) exhibited similar expression profiles among various tissues (Fig 3). During fiber development, similar expression profiles of homoeologous genes occurred for *GobHLH064s* and *GobHLH123s*, but not for *GobHLH110s* and *GobHLH130s* (Fig 4). *GobHLH110A* only showed significant expression in 0-DPA ovules, while *GobHLH110D* was also highly expressed in late-stage fibers (20DPA). *GobHLH130A* had relatively high expression levels at early stage (0 and 5DPA), while high expression levels of *GobHLH130D* was maintained from 5 to 15 DPA. These data show that the homoeologous genes of different genomes may express differentially in tetraploid cottons, especially in developing fibers.

## Discussion

It is rather difficult to thoroughly identify bHLH proteins or coding genes in a certain genome. Traditionally, one or several bHLH sequences were employed as probe to search homologs in sequenced genomes. Due to the complexity and diversity of bHLH families, this method generally resulted in missing of some members. For example, different probe sequences used in homolog searching detected partially-overlapped sets of bHLH proteins in *Arabidopsis* [13–16,48]. In this study, a total of 35 probe sequences, including representative sequences of 32 plant bHLH subfamilies and three *Arabidopsis* orphans determined by Carretero-Paulet et al [13], were used to search homologs in sequenced cotton and cacao genomes. These probe sequences represented a much broader set of bHLH domains, and might cover most diversity in plant bHLH domains. Finally, we identified 272, 255, and 256 distinct bHLH genes from Gr-JGI, Gr-CGP, and Ga-CGP, respectively. In contrast, Cottongen detected less bHLH genes (258 and 243 in Gr-JGI and Gr-CGP, respectively) by using a bHLH consensus (IPR011598) as searching probe [38]. On the other hand, the bHLH domains determined in this study (except TcbHLH087) were all clustered along with plant reference bHLHs, indicating that multiple sequence alignment excluded false positives efficiently. These data suggest that searching with diverse probes and re-confirming by multiple sequence alignment may facilitate comprehensive detection of homologs in a certain genome.

Genome sequences are the major sources to systematically detect protein family members, and most of cotton bHLH genes are identified from sequenced genomes (Table 1; S3 Table). However, the genome sequencing projects in cottons are still in infancy compared to those in model plants, and currently available cotton genome sequences are relatively fragmented [18,33,34]. Consequently, only a part of the 'complete' set of cotton bHLH genes could be detected in a single sequenced genome (Table 1; S3 Table). For example, GobHLH130 was first identified only in Gr-JGI (D-genome). But cloning and PCR detection showed that a full-length *GobHLH130A* gene did exist in tetraploid cottons and A-genome donor *G*. *arboreum*, indicating that the corresponding sequences were not correctly assembled or annotated in Gr-CGP and Ga-CGP. Therefore, it is reasonable currently to integrate all the bHLHs detected in different sources to constitute the cotton bHLH reference genes.

Classification of plant bHLH proteins varied in different studies, probably due to different methods and sequences adopted [12–16,48]. A genome-wide classification of bHLH family in *Arabidopsis*, poplar, rice, moss, and algae assigned 638 bHLHs into 32 subfamilies [13]. In this study, the bHLHs from *Arabidopsis*, cotton, cacao, and some representatives from other plants were clustered into 30 subfamilies, a little less than those determined by Carretero-Paulet et al [13]. In our classification, S5 and S12 were divided into two subfamilies (S5a and S5b, S12a and S12b, respectively; Fig 1; S2 Fig), and S18, S19, S20, S21, and S22 were merged into a single group (S18). This difference may be attributed to different methods and less sequences used in our study. Nevertheless, most subfamilies in our classification are consistent with the previous system [13]. S5 and S12 subfamilies in Carretero-Paulet’s system are also divided into two subgroups [13], corresponding to the separate subfamilies (S5a and S5b, S12a and S12b, respectively) in our classification. Meanwhile, the bHLH proteins of the present S18 subfamily (including S18-S22 in Carretero-Paulet’s system) share similar structures, i.e., they are short in length, and contain little conserved domains other than bHLH domain (S6 Table) [13,46]. Taken together, the classification of bHLH proteins in our study is comparable to the previous system [13], and lays a good foundation for exploring the evolutionary characteristics of cotton bHLH family.

The bHLH protein family expanded significantly in higher plants and formed one of the largest transcription factor families [9,12,13]. In this study, we identified a total of 289 cotton reference bHLH genes from three independent cotton genome sequencing projects. This may be very close to the number of complete bHLH genes in a single cotton genome, although small changes in this data can’t be excluded. Considered that the coding genes are doubled and basically maintained after tetraploidization (Fig 2), bHLH family in tetraploid cottons may harbor around 580 genes. Expression analyses suggest that most of bHLH genes in tetraploids may express in certain tissues (Figs 3 and 4). Although cotton bHLHs are clustered into the similar subfamilies as *Arabidopsis* and cacao, multiple copies of bHLH genes may increase the complexity in regulation and also the possibility of mutation, neofunctionalization, and subfunctionalization in cotton species [1,49].

Retrotransposons comprise a large part of genomes in higher plants [18,33,34,50,51]. However, little is known about their biological function and evolutionary effects in cotton genomes. Recently, Woodhouse et al indicated that transponson-derived small RNA might induce differential silencing of homoeologous genes from different subgenomes and lead to genome dominance in hexaploid *Brassica rapa* [27]. In this study, we revealed that a LTR retrotransposon inserted in *GhbHLH062A* gene eliminated the transcription of this gene in tetraploid cotton. Interestingly, this retrotransposon may originate from D-genome progenitor, for it shares high sequence similarity with some LTR retrotransposons from *G*. *ramondii* rather those from *G*. *arboreum* (data not shown). Furthermore, this retrotransposon insertion can be detected both in *G*. *hirsutum* and *G*. *barbadense* (S4 Fig), suggesting that retrotransposon duplication and insertion may be a common event during tetraploidization in cotton. It may be valuable to elucidate the evolutionary and genetic effects of retrotransposon duplication in tetraploid cottons.

New allopolyploids harbor divergent genomes of their progenitors, and thus entail extensive genetic and epigenetic changes, including gene deletion, recombination, gene conversion, and varied expression, to conciliate different sets of genetic materials [18,24,28,29,31,52]. Nevertheless, recent genome sequencing researches indicated that, in oilseed and cotton, most of orthologous genes from progenitors maintained as homoeologs in allotetraploid, and expressed in certain tissues [18,28]. Consistent with previous reports [18,24,30], all the S5a and S5b bHLH genes remained in upland cotton. Except *GhbHLH062A* which was disrupted by retrotransposon insertion, all the rest genes expressed significantly in upland cotton. Interestingly, two pairs of homoeologous bHLH genes (*GobHLH064A* and *D*, *GobHLH130A* and *D*) had different, but complementary expression profiles during fiber development. The A-genome genes expressed predominantly at the early stages (<10DPA), while D-genome ones mainly at the late stages (>10DPA; Fig 4). S5 bHLHs in *Arabidopsis* function as important regulator of trichome differentiation and flavonoid biosynthesis [4,46,47]. We envision that the complementary expression of S5a and S5b bHLH homoeologs may play a role in promoting fiber development in allotetraploid cottons.

## Supporting Information

S1 Fig Alignment of *Arabidopsis*, *Gossypium*, and *Theobroma* bHLH domains. A total of 605 bHLH domains (169 from A. thaliana, 289 from *Gossypium*, 139 from *T*. *cacao*, four from *P*. *patens*, two from *C*. *reinhardtii* and two from *O*. *sativa*) were aligned using AlignX program in software Vector NTI (Invitrogen) with default parameters. The consensus sequence is indicated at the bottom. The amino acid residuals conserved in over 80% and 60% sequences are shaded in light blue and green, respectively.
(TIF)

S2 Fig Phylogenetic tree of *Arabidopsis*, *Gossypium*, and *Theobroma* bHLH domains. The phylogenetic trees were constructed and tested using NJ, ML, and MP methods on the basis of the alignment shown in S1 Fig. The NJ tree is presented, and subfamilies are indicated by square brackets with bootstraps (%) supported in NJ, ML and MP test (-, not supported). The scale bar indicates the estimated number of amino acid replacement per site. The sequences from *A*. *thaliana* (At), *Gossypium* (Go) and *T*. *cacao* (Tc) are marked with solid dots, open circles and squares, respectively.
(PDF)

S3 Fig Alignment of S5a and S5b bHLH proteins from *Gossypium* and *A*. *thaliana*. The S5a and S5b bHLH proteins from *G*. *hirsutum* (Gh) and the diploid progenitors *G*. *arboreum* (Ga) and *G*. *raimondii* (Gr) are aligned with *Arabidopsis* homologous proteins. The amino acid residuals conserved in 100%, over 80% and 60% sequences are shaded in black, dark grey and light grey, respectively. The conserved domains identified by Carretero-Paulet et al (2010) in S5 bHLH subfamily are marked by black bars, and red bar indicates the bHLH domains.
(TIF)

S4 Fig Alignment of *GobHLH062* sequences around the LTR insertion site. The sequences are from *GobHLH062D* of *G*. *hirsutum* and *G*. *raimondii*, *GobHLH062A* of *G*. *arboreum*, *G*. *hirsutum* Yumian No.1 (-Y) and T586 (-T), and *G*. *barbadense*. Intron, exon, and LTR retrotransposon sequences are marked by black lines, blue and red bars, respectively. Identical sequences are shaded in grey. Dashes indicate gaps in the alignment, while dots represent the omitted LTR sequences.
(TIF)

S5 Fig Alignment of *GobHLH130* sequences around the deletion site. The sequences are from *GobHLH130A* of *G*. *hirsutum* and *G*. *arboreum*, *GobHLH130D* of *G*. *raimondii*, *G*. *hirsutum* Yumian No.1 (-Y) and T586 (-T), and *G*. *barbadense*. Identical and conserved (>60%) sequences are shaded in light blue and pink, respectively. Dashes indicate the deleted sequences in *GhbHLH130D*.
(TIF)

S1 Table Internet sources of cotton bHLH sequences. (XLSX)

S2 Table The probe sequences used for searching bHLH protein. (XLSX)

S3 Table Coding of *Gossypium* bHLH reference genes and the corresponding sequences in different sources. (XLSX)

S4 Table Plant bHLH proteins used in phylogenetic analysis from species other than *Gossypium*. (XLSX)

S5 Table Primers used in this study. (XLSX)

S6 Table Phylogenetic classification, conserved domains, and known biological functions of bHLH proteins from *Arabidopsis*, *Theobroma* and *Gossypium*. (DOC)
