# Supplementary figures and images for: The Basic/Helix-Loop-Helix Protein Family in Gossypium: Reference Genes and Their Evolution during Tetraploidization
Source: PLoS One. 2015 May 18;10(5):e0126558. doi: 10.1371/journal.pone.0126558 (PMC4436304; doi:10.1371/journal.pone.0126558)

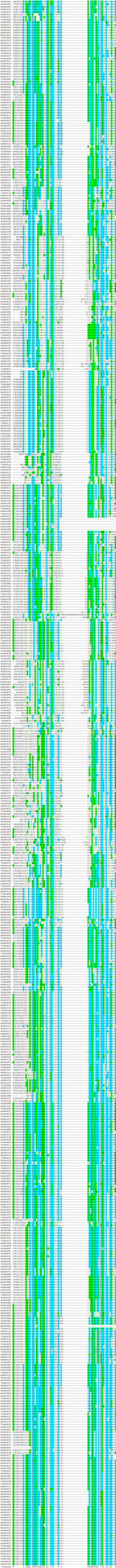

Supplement: S1 Fig — A total of 605 bHLH domains (169 from A. thaliana, 289 from Gossypium, 139 from T. cacao, four from P. patens, two from C. reinhardtii and two from O. sativa) were aligned using AlignX program in software Vector NTI (Invitrogen) with default parameters. The consensus sequence is indicated at the bottom. The amino acid residuals conserved in over 80% and 60% sequences are shaded in light blue and green, respectively. (TIF) [file pone.0126558.s001.tif]

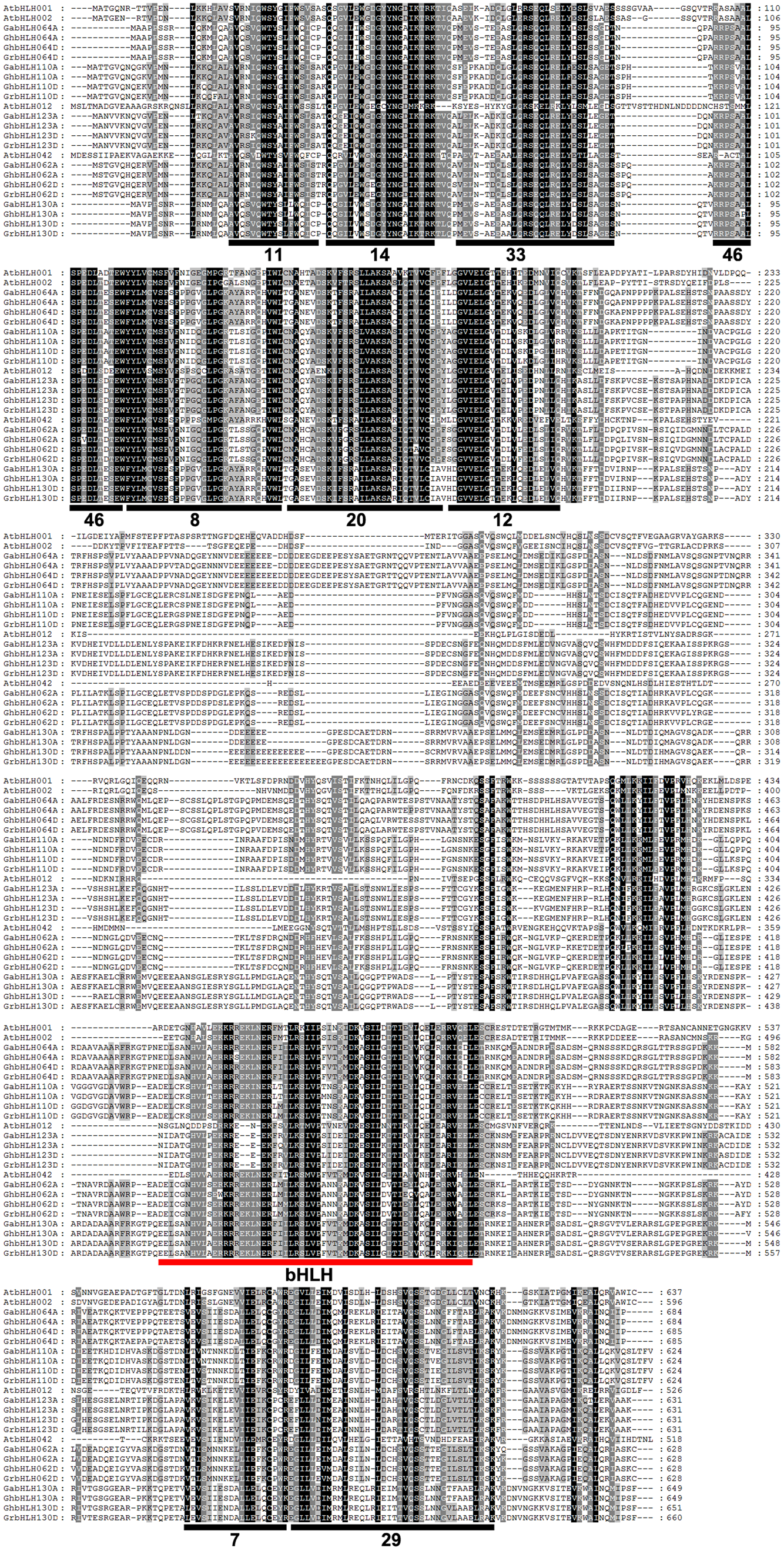

Supplement: S3 Fig — The S5a and S5b bHLH proteins from G. hirsutum (Gh) and the diploid progenitors G. arboreum (Ga) and G. raimondii (Gr) are aligned with Arabidopsis homologous proteins. The amino acid residuals conserved in 100%, over 80% and 60% sequences are shaded in black, dark grey and light grey, respectively. The conserved domains identified by Carretero-Paulet et al (2010) in S5 bHLH subfamily are marked by black bars, and red bar indicates the bHLH domains. (TIF) [file pone.0126558.s003.tif]

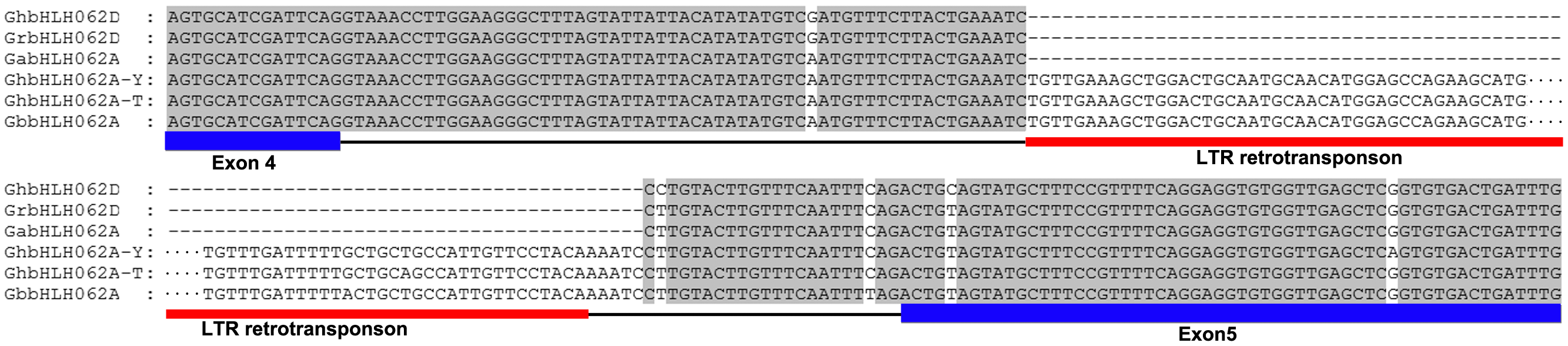

Supplement: S4 Fig — The sequences are from GobHLH062D of G. hirsutum and G. raimondii, GobHLH062A of G. arboreum, G. hirsutum Yumian No.1 (-Y) and T586 (-T), and G. barbadense. Intron, exon, and LTR retrotransposon sequences are marked by black lines, blue and red bars, respectively. Identical sequences are shaded in grey. Dashes indicate gaps in the alignment, while dots represent the omitted LTR sequences. (TIF) [file pone.0126558.s004.tif]

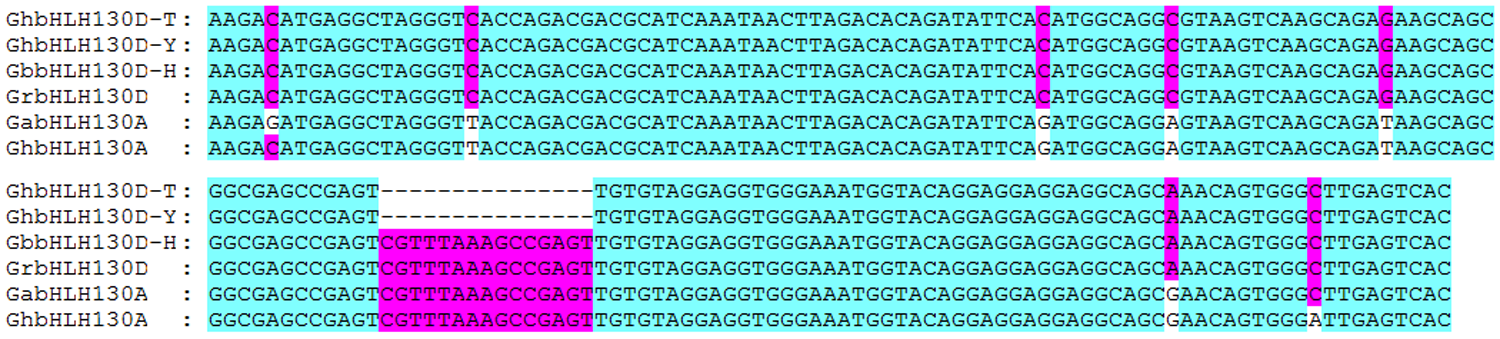

Supplement: S5 Fig — The sequences are from GobHLH130A of G. hirsutum and G. arboreum, GobHLH130D of G. raimondii, G. hirsutum Yumian No.1 (-Y) and T586 (-T), and G. barbadense. Identical and conserved (>60%) sequences are shaded in light blue and pink, respectively. Dashes indicate the deleted sequences in GhbHLH130D. (TIF) [file pone.0126558.s005.tif]
