# Supplementary material for: The Basic/Helix-Loop-Helix Protein Family in Gossypium: Reference Genes and Their Evolution during Tetraploidization
Source: PLoS One. 2015 May 18;10(5):e0126558. doi: 10.1371/journal.pone.0126558 (PMC4436304; doi:10.1371/journal.pone.0126558)
Supplement: S6 Table — (DOC) [file pone.0126558.s011.doc]

***S6 Table. Phylogenetic classification, conserved domains and known biological functions of bHLH proteins from Arabidopsis, Theobroma and Gossypium***

| Subfamily | Arabidopsis bHLHs | *Gossypium* bHLHs | Theobroma bHLHs | Conserved domains | Arabidopsis mutants and gene functions |
| --- | --- | --- | --- | --- | --- |
| **S1** | AtbHLH021  AtbHLH022  AtbHLH027  AtbHLH029  AtbHLH033  AtbHLH035  AtbHLH061  AtbHLH090  AtbHLH093  AtbHLH116 | GobHLH023  GobHLH033  GobHLH044  GobHLH050  GobHLH095  GobHLH117  GobHLH121  GobHLH141  GobHLH153  GobHLH163  GobHLH176  GobHLH184  GobHLH196  GobHLH203  GobHLH211  GobHLH222  GobHLH271 | TcbHLH008  TcbHLH030  TcbHLH044  TcbHLH060  TcbHLH073  TcbHLH106  TcbHLH117  TcbHLH127  TcbHLH131 | 2-1-4 | [ice1](http://www.arabidopsis.org/servlets/TairObject?type=germplasm&id=1005959039), Reduction in plant chilling and freezing tolerance (AtbHLH033)  fit1, Defects in Fe uptake (AtbHLH029)  [ICE2, Cold acclimation](http://www.arabidopsis.org/servlets/TairObject?type=publication&id=501729093) (AtbHLH035)  dyt1, Defects in the tapetum at anther stage 4 and later (AtbHLH022)  ams, Sporophytic male sterile (AtbHLH021) |
| **S2** | AtbHLH003  AtbHLH004  AtbHLH005  AtbHLH006  AtbHLH013  AtbHLH014  AtbHLH017  AtbHLH028 | GobHLH011  GobHLH051  GobHLH057  GobHLH059  GobHLH061  GobHLH103  GobHLH124  GobHLH125  GobHLH132  GobHLH161  GobHLH171  GobHLH172 | TcbHLH024  TcbHLH025  TcbHLH065  TcbHLH068 | 11-14-34-8-20-12-10-2-1-7-4 | MYC4, JAZ-interacting activator of JA-responses (AtbHLH004)  MYC3, JAZ-interacting activator of JA-responses (AtbHLH005)  MYC2, JAZ-interacting activator of JA-responses (AtbHLH006)  bhlh3 bhlh13 bhlh14 bhlh17, increased JA sensitivity (AtbHLH003, AtbHLH013, AtbHLH014 and AtbHLH017)  [ataib](http://www.arabidopsis.org/servlets/TairObject?type=germplasm&id=5016227168), Weak insensitivity to ABA (AtbHLH013)  atnig1-1, Reduction in resistance to salt stress (AtbHLH017) |
| **S3** | AtbHLH018  AtbHLH019  AtbHLH020  AtbHLH025 | GobHLH035  GobHLH042  GobHLH043  GobHLH118  GobHLH139  GobHLH140  GobHLH159  GobHLH254 | TcbHLH006  TcbHLH007 | 2-1-7-4 | ER development; response to endophytic fungus (AtbHLH020) |
| **S4** | AtbHLH011  AtbHLH034  AtbHLH047  AtbHLH104  AtbHLH105  AtbHLH115  AtbHLH121 | GobHLH024  GobHLH028  GobHLH039  GobHLH087  GobHLH114  GobHLH136  GobHLH148  GobHLH191  GobHLH239  GobHLH268 | TcbHLH001  TcbHLH031  TcbHLH038  TcbHLH056  TcbHLH086  TcbHLH107  TcbHLH108 | 31-2-1-9-15 | PYE, Regulator of response to iron deficiency (AtbHLH047)  ILR3, conjugated IAA metabolism (AtbHLH105) |
| **S5a** | AtbHLH001  AtbHLH002  AtbHLH012 | GobHLH062  GobHLH110  GobHLH123 | TcbHLH026  TcbHLH071 | 11-14-33-46-8-20-12-2-1-7-29 | [gl3-1](http://www.arabidopsis.org/servlets/TairObject?type=polyallele&id=115170), trichomes unbranched and reduced in number (AtbHLH001)  [egl3-1](http://www.arabidopsis.org/servlets/TairObject?type=polyallele&id=502795326), reduced trichomes, anthocyanin, and seed coat mucilage and abnormally patterned stomata. (AtbHLH002)  [myc1-1](http://www.arabidopsis.org/servlets/TairObject?type=polyallele&id=49147), Increased ectopic root-hair cells (AtbHLH012) |
| **S5b** | AtbHLH042 | GobHLH064  GobHLH130 | TcbHLH020 |  | tt8-1, absence of brown pigment in seed coat (testa) (AtbHLH042) |
| **S7** | AtbHLH041  AtbHLH092 | GobHLH002  GobHLH073  GobHLH106  GobHLH178  GobHLH214 | TcbHLH085  TcbHLH123 |  |  |
| **S8** |  | GobHLH066  GobHLH070  GobHLH091  GobHLH187  GobHLH219  GobHLH226  GobHLH233  GobHLH249 | TcbHLH072  TcbHLH132 |  |  |
| **S9** | AtbHLH010  AtbHLH089  AtbHLH091  AtbHLH138 | GobHLH220  GobHLH221  GobHLH248 | TcbHLH133 | 2-1-7-29 |  |
| **S10** | AtbHLH045  AtbHLH057  AtbHLH067  AtbHLH070  AtbHLH071  AtbHLH094  AtbHLH096  AtbHLH097  AtbHLH098  AtbHLH099 | GobHLH045  GobHLH068  GobHLH075  GobHLH092  GobHLH104  GobHLH126  GobHLH127  GobHLH145  GobHLH170  GobHLH173  GobHLH199  GobHLH230  GobHLH237  GobHLH269 | TcbHLH010  TcbHLH023  TcbHLH064  TcbHLH067  TcbHLH083  TcbHLH084  TcbHLH096  TcbHLH115 | 28-2-26-1-7-4-19 | SPCH, necessary and sufficient for the asymmetric divisions that establish the stomatal lineage (AtbHLH099)  mtp1-1, Enhanced sensitivity to elevated Zn (AtbHLH071)  Mute, Complete absence of stomata (AtbHLH045)  fama-1, Failed differentiation of guard cells (AtbHLH098) |
| **S11** | AtbHLH095 | GobHLH032  GobHLH260  GobHLH283 | TcbHLH034  TcbHLH112 |  | zou, retarded embryo growth |
| **S12a** | AtbHLH038  AtbHLH039  AtbHLH100  AtbHLH101  AtbHLH160 | GobHLH097  GobHLH150  GobHLH182  GobHLH259 | TcbHLH033  TcbHLH092  TcbHLH129 | 2-1-41 | ORG2 (AtbHLH038), ORG3 (AtbHLH039), response to iron ion, response to SA |
| **S12b** | AtbHLH036  AtbHLH055  AtbHLH118  AtbHLH120  AtbHLH125  AtbHLH126  AtbHLH162 | GobHLH004  GobHLH076  GobHLH102  GobHLH162  GobHLH174  GobHLH175  GobHLH189  GobHLH218  GobHLH224  GobHLH241  GobHLH253  GobHLH255  GobHLH281  GobHLH282  GobHLH285 | TcbHLH040  TcbHLH042  TcbHLH043  TcbHLH052  TcbHLH061  TcbHLH062  TcbHLH063  TcbHLH069  TcbHLH070  TcbHLH104  TcbHLH136  TcbHLH137 | 2-1-34-4 |  |
| **S13** | AtbHLH030  AtbHLH032  AtbHLH051  AtbHLH106  AtbHLH107  AtbHLH131 | GobHLH119  GobHLH149  GobHLH167  GobHLH188  GobHLH197  GobHLH225  GobHLH243  GobHLH247  GobHLH262  GobHLH280  GobHLH284  GobHLH287  GobHLH288  GobHLH289 | TcbHLH009  TcbHLH012  TcbHLH050  TcbHLH091  TcbHLH103  TcbHLH134 | 35-2-1-25-4 | bhlh32, expression of PPCK is elevated under Pi starvation. Under high levels of Pi, root hair contained significantly more total Pi and more anthocyanin than the wild-type. DFR expression in Pi-sufficient conditions was substantially increased. |
| **S14** | AtbHLH046  AtbHLH102  AtbHLH141 | GobHLH014  GobHLH108  GobHLH115  GobHLH165  GobHLH202  GobHLH264 | TcbHLH048  TcbHLH058  TcbHLH077 | 2-1-50-4 | BIM (BES1-INTERACTING MYC-LIKE)1 ( AtbHLH046), synergistically interacts with BES1 to bind to E box sequences (CANNTG). Positively modulates the shade avoidance syndrome in Arabidopsis seedlings.  BIM3 (AtbHLH141) and BIM2 (AtbHLH102), PAR1 (PHYTOCHROME RAPIDLY REGULATED 1)-interacting proteins that positively modulates the shade avoidance syndrome in Arabidopsis seedlings. |
| **S15** | AtbHLH068  AtbHLH103  AtbHLH110  AtbHLH111  AtbHLH112  AtbHLH113  AtbHLH114  AtbHLH123  AtbHLH133  AtbHLH153  AtbHLH154 | GobHLH001  GobHLH027  GobHLH040  GobHLH071  GobHLH079  GobHLH094  GobHLH156  GobHLH177  GobHLH185  GobHLH190  GobHLH192  GobHLH205  GobHLH206  GobHLH213  GobHLH217  GobHLH223  GobHLH229  GobHLH234  GobHLH242  GobHLH257  GobHLH261  GobHLH286 | TcbHLH135  TcbHLH080  TcbHLH153  TcbHLH154  TcbHLH046  TcbHLH002  TcbHLH035  TcbHLH105  TcbHLH109  TcbHLH114  TcbHLH124 | 2-1-13 | ERP (AtbHLH154) :Response to ethylene and gibberellin stimulus |
| **S16** | AtbHLH134  AtbHLH135  AtbHLH136  AtbHLH161  AtbHLH163  AtbHLH164 | GobHLH021  GobHLH022  GobHLH048  GobHLH077  GobHLH089  GobHLH090  GobHLH112  GobHLH113  GobHLH166  GobHLH168  GobHLH201  GobHLH246  GobHLH263 | TcbHLH049  TcbHLH054  TcbHLH055  TcbHLH074  TcbHLH075 | 2-1 | AtbHLH135, ATBS1(activation-tagged bri1(brassinosteroid-insensitive 1)-suppressor 1), TMO7, target of monopteros 7, PRE3, paclobutrazol resistance 3.  BNQ1(AtbHLH136), BNQ2 (AtbHLH134, PRE2) and BNQ3( AtbHLH161) directly and negatively regulated by AP3 and PI in petals, required for appropriate regulation of flowering time.  AtbHLH136, PRE1 and IBH1 form a pair of antagonistic HLH/bHLH transcription factors that function downstream of BZR1 to mediate brassinosteroid regulation of cell elongation.  bnq3, sepals and carpels are pale yellow or white,while the inflorescence stems and siliques are purple. Floral organs are smaller than WT. Flowers, cauline leaves, stems, and siliques have a decreased amount of chlorophyll as compared to WT.  AtbHLH163 (KDR, PRE6), involved in blue/far-red light signaling. Physically interacts with HFR1 and negatively regulates its activity. |
| **S17** | AtbHLH142  AtbHLH143  AtbHLH144  AtbHLH145 | GobHLH084  GobHLH186  GobHLH198  GobHLH200  GobHLH212  GobHLH227 | TcbHLH037  TcbHLH102  TcbHLH116 | 26-2-1 | sac51-d, Upregulation of SAC51 [suppressor of acaulis 51] ( AtbHLH142) reverses the dwarf phenotype caused by a loss-of-function in ACL5 gene encoding spermine synthase. |
| **S18** | AtbHLH146  AtbHLH147  AtbHLH148  AtbHLH149  AtbHLH150  AtbHLH151  AtbHLH158  AtbHLH159  AtbHLH165  AtbHLH166  AtbHLH167  AtbHLH168  AtbHLH169  AtbHLH170 | GobHLH016  GobHLH025  GobHLH026  GobHLH031  GobHLH034  GobHLH056  GobHLH067  GobHLH069  GobHLH074  GobHLH080  GobHLH107  GobHLH120  GobHLH135  GobHLH146  GobHLH164  GobHLH204  GobHLH207  GobHLH238  GobHLH240  GobHLH245  GobHLH250  GobHLH256  GobHLH266  GobHLH270  GobHLH272  GobHLH273  GobHLH274  GobHLH275 | TcbHLH013  TcbHLH051  TcbHLH045  TcbHLH076  TcbHLH125  TcbHLH015  TcbHLH126  TcbHLH097  TcbHLH088 | 2-1 | AtbHLH158, ILI1binding bHLH 1 (IBH1) .  AtbHLH150, AtBS1(activation-tagged BRI1 suppressor 1)-interacting factor 1 (AIF1).  AtbHLH148, AIF2.  AtbHLH147, AIF3.  AtbHLH149, AIF4.  AtbHLH167, PIR1. AtbHLH159, PIR2. AtbHLH168, PIR3.  AtbHLH165, PHYTOCHROME RAPIDLY REGULATED1 (PAR1), and AtbHLH166 (PAR2), Up regulated after simulated shade perception. Acts in the nucleus to control plant development and as a negative regulator of shade avoidance response. Functions as transcriptional repressor of auxin-responsive genes SAUR15 and SAUR68. |
| **S23** | AtbHLH155  AtbHLH156  AtbHLH157 | GobHLH020  GobHLH038  GobHLH047  GobHLH054  GobHLH058  GobHLH096  GobHLH100  GobHLH183 | TcbHLH018  TcbHLH066  TcbHLH090  TcbHLH130 | 11-36-12-2-1-6 | AtbHLH151, UPBEAT1 (UPB1), regulates the expression of a set of peroxidases that modulate the balance of reactive oxygen species (ROS) between the zones of cell proliferation and the zone of cell elongation where differentiation begins. Disruption of UPB1 activity alters this ROS balance, leading to a delay in the onset of differentiation. Root development. upb1-1, longer root than the wild type; significant increase in cortex cell number indicating enlargement of the meristem.  AtbHLH155, conserved peptide upstream open reading frame 7 (CPUORF7).  AtbHLH156 (LHW), promotes the production of stele cells in root meristems and is required to establish and maintain the normal vascular cell number and pattern in primary and lateral roots. lhw-1, reduced root vascular population. Roots lose bilateral symmetry and are monarch instead of diarch. |
| **S24** | AtbHLH008  AtbHLH009  AtbHLH015  AtbHLH016  AtbHLH023  AtbHLH024  AtbHLH026  AtbHLH056  AtbHLH065  AtbHLH072  AtbHLH073  AtbHLH109  AtbHLH119  AtbHLH124  AtbHLH127  AtbHLH132 | GobHLH007  GobHLH010  GobHLH012  GobHLH018  GobHLH049  GobHLH088  GobHLH122  GobHLH128  GobHLH133  GobHLH138  GobHLH147  GobHLH158  GobHLH160  GobHLH194  GobHLH252 | TcbHLH005  TcbHLH014  TcbHLH017  TcbHLH022  TcbHLH027  TcbHLH057  TcbHLH098  TcbHLH111 | 44-16-2-1 | AtbHLH015, PIF1, a key negative regulator of phytochrome-mediated seed germination and acts by inhibiting chlorophyll biosynthesis, light-mediated suppression of hypocotyl elongation and far-red light-mediated suppression of seed germination, and promoting negative gravitropism in hypocotyls. Light reduces this activity in a phy-dependent manner. The protein preferentially interacts with the Pfr forms of Phytochrome A (PhyA) and Phytochrome B (PhyB), is physically associated with APRR1/TOC1 and is degraded in red (R) and far-red (FR) light through the ubiquitin (ub)-26S proteasome pathway to optimize photomorphogenic development in Arabidopsis. It also negatively regulates GA3 oxidase expression. pif1-1, bleaching phenotype of the seedlings grown for 5 days in the dark and then transferred to white light due to the overaccumulation of chlorophyll precursor, photochlorophyllide.  AtbHLH009, PIF4, interacts with active PhyB protein. Negatively regulates phyB mediated red light responses. Involved in shade avoidance response. Protein abundance is negatively regulated by PhyB. pif4-2, hypocotyl hypersensitivity to red light.  AtbHLH065, PIL6, physically associated with APRR1/TOC1 and is a member of PIF3 transcription factor family. Involved in shade avoidance. Functions as negative regulator of PhyB. Protein levels are modulated by phytochrome B. pil6-1, seedlings are hypersensitive to red light, displaying shorter hypocotyls and larger cotyledons than the wild type. They also exhibit hypersensitivity to red light in hook opening and cotyledon separation.  AtbHLH072, PIF7, interacts specifically with the far-red light-absorbing Pfr form of phyB through a conserved domain called the active phyB binding motif. Upon light exposure, PIF7 rapidly migrates to intranuclear speckles, where it colocalizes with phyB. Role as negative regulator of phyB-mediated seedling deetiolation.  AtbHLH016, unfertilized embryo sac 10 (UNE10);  AtbHLH124 (PIL1), AtbHLH132(PIL2), physically associated with APRR1/TOC1 and are members of PIF3 transcription factor family. pil1, seedlings exhibit a significant shift (~6 h) in the phase of the circadian rhythm of hypocotyl elongation responses to low R/FR.  AtbHLH008 (PIF3), interacting with photoreceptors phyA and phyB. Forms a ternary complex in vitro with G-box element of the promoters of LHY, CCA1. Acts as a negative regulator of phyB signalling. Binds to anthocyanin biosynthetic genes in a light- and HY5-independent fashion. Regulation of anthocyanin, regulation of seed germination, gibberellic acid signaling, regulation of chlorophyll metabolism, negative gravitropism. pif3-7, Shorter hypocotyls than the wild type when grown under red light. Delayed chlorophyll accumulation.  AtbHLH024 (SPT), spt-2, gynoecium flattened medially at apex, sometimes unfused at apex, silique shorter, flattened laterally at apex, style and stigma reduced, transmitting tract absent, reduced seed set.  AtbHLH073, ALCATRAZ(ALC), involved in fruit dehiscence. Mutant siliques fail to dehisce.  AtbHLH026, HFR1 (long hypocotyl in far-red) involved in phytochrome signaling. Mutants exhibit a long-hypocotyl phenotype only under far-red light but not under red light and are defective in other phytochrome A-related responses. Mutants also show blue light response defects. HFR1 interacts with COP1, co-localizes to the nuclear specks and is ubiquinated by COP1. hfr1, Long hypocotyl in far-red light |
| **S25** | AtbHLH031  AtbHLH044  AtbHLH048  AtbHLH049  AtbHLH050  AtbHLH058  AtbHLH060  AtbHLH062  AtbHLH063  AtbHLH064  AtbHLH074  AtbHLH075  AtbHLH076  AtbHLH077  AtbHLH078  AtbHLH079  AtbHLH137 | GobHLH005  GobHLH006  GobHLH009  GobHLH013  GobHLH015  GobHLH017  GobHLH030  GobHLH037  GobHLH063  GobHLH072  GobHLH078  GobHLH081  GobHLH086  GobHLH093  GobHLH098  GobHLH101  GobHLH105  GobHLH109  GobHLH111  GobHLH116  GobHLH134  GobHLH142  GobHLH151  GobHLH152  GobHLH154  GobHLH155  GobHLH157  GobHLH169  GobHLH210  GobHLH215  GobHLH232  GobHLH236  GobHLH244  GobHLH258  GobHLH265  GobHLH276  GobHLH277  GobHLH278  GobHLH279 | TcbHLH003  TcbHLH016  TcbHLH032  TcbHLH041  TcbHLH047  TcbHLH053  TcbHLH059  TcbHLH079  TcbHLH082  TcbHLH093  TcbHLH118  TcbHLH138  TcbHLH139 | 3-2-21-1-23 | AtbHLH063, CIB1, interacts with CRY2 (cryptochrome 2) in a blue light-specific manner in yeast and Arabidopsis cells, and it acts together with additional CIB1-related proteins to promote CRY2-dependent floral initiation. CIB1 positively regulates FT expression. cib1cib5 double mutant showed a mild but statistically significant delay of flowering under a photoperiodic inductive condition for 4 days, and removed back to short-day to continue grow until flowering.  AtbHLH058, a brassinosteroid signaling component BEE2 (BR-ENHANCED EXPRESSION 2). Positively modulates the shade avoidance syndrome in Arabidopsis seedlings.  AtbHLH064, HBI1(homolog of BEE2 interacting with ibh 1)  AtbHLH044, AtbHLH050, brassinosteroid signaling components BEE2 and BEE3. Positively modulates the shade avoidance syndrome in Arabidopsis seedlings.  AtbHLH075, CESTA, a positive regulator of brassinosteroid biosynthesis. AtbHLH076, CIB5 (cryptochrome-interacting basic-helix-loop-helix), interacts with CRY2 and forms heterodimer with CIB1 in vitro. Regulates flowering time redundantly with CIB1.  AtbHLH031, BIGPETAL, BPE, involved in the control of petal size. BPE is expressed via two mRNAs derived from an alternative splicing event. The BPEub (AT1G59640.1) transcript is expressed ubiquitously, whereas the BPEp (AT1G59640.2) transcript is preferentially expressed in petals. Plants that lack the petal-expressed variant BPEp have larger petals as a result of increased cell size. BPEp is positively regulated downstream of APETALA3, PISTILLATA, APETALA1 and PISTILLATA3 and is negatively regulated downstream of AGAMOUS. bigpetal-1, •Larger petal size as a result of increased petal cell size. |
| **S26** | AtbHLH007  AtbHLH059  AtbHLH066  AtbHLH069  AtbHLH082 | GobHLH003  GobHLH019  GobHLH055  GobHLH065  GobHLH129  GobHLH131  GobHLH143  GobHLH144  GobHLH179  GobHLH180  GobHLH216 | TcbHLH019  TcbHLH021  TcbHLH095  TcbHLH121  TcbHLH122 | 3-2-1-30-5 | AtbHLH066 (AtLRL1), AtbHLH069 (AtLRL2), and AtbHLH082 (AtLRL3), Arabidopsis homologs of the Lotus japonicus ROOTHAIRLESS1 (LjRHL1) gene.  AbHLH059, unfertilized embryo sac 12 (UNE12). |
| **S27** | AtbHLH080  AtbHLH081  AtbHLH122  AtbHLH128  AtbHLH129  AtbHLH130 | GobHLH036  GobHLH052  GobHLH060  GobHLH099  GobHLH181  GobHLH235  GobHLH267 | TcbHLH028  TcbHLH081  TcbHLH094  TcbHLH120  TcbHLH128 | 22-3-2-43-1 | AtbHLH081, FBH2 (flowering bhlh 2).  AtbHLH122, FBH3(flowering bhlh 3), AKS1(aba-responsive kinase substrate 1), involved in photoperiodism flowering.  AtbHLH130, FBH4(flowering bhlh 3); AKS3(aba-responsive kinase substrate 3). |
| **S28** | AtbHLH054  AtbHLH083  AtbHLH084  AtbHLH085  AtbHLH086  AtbHLH139 | GobHLH008  GobHLH053  GobHLH085  GobHLH195  GobHLH219 | TcbHLH036  TcbHLH078  TcbHLH119 | 3-2-1 | AtbHLH84, RSL3.  AtbHLH085, RSL2(root hair defective 6-like 2), expressed concurrently with RSL4 and its expression was controlled by RHD6 and RSL1. Required for root-hair growth.  AtbHLH054, RSL4, sufficient to promote postmitotic cell growth in root-hair cells, is a direct transcriptional target of RHD6.  AtbHLH083, ROOT HAIR DEFECTIVE 6 (RHD6). rhd6-1, altered root hair initiation; reduced number of root hairs; overall basal shift in the site of root hair emergency; relatively high frequency of epidermal cells with multiple root hairs; abnormal root hair phenotype is suppressed by ethylene or auxin..  AtbHLH86, RHD SIX-LIKE 1 (RSL1). |
| **S30** | AtbHLH052  AtbHLH053  AtbHLH117 | GobHLH029  GobHLH082  GobHLH083 | TcbHLH039  TcbHLH099  TcbHLH110 | 2-1 |  |
| **S31** | AtbHLH037  AtbHLH040  AtbHLH043  AtbHLH087  AtbHLH088  AtbHLH140 | GobHLH041  GobHLH046  GobHLH137  GobHLH193  GobHLH208  GobHLH228  GobHLH231  GobHLH251 | TcbHLH004  TcbHLH011  TcbHLH101  TcbHLH113  TcbHLH140 | 18-10-2-1 | AtbHLH88, HECATE 1 (HEC1), AtbHLH37 (HEC2), AtbHLH43, HEC3), involved in ovary septum development, transmitting tissue development, carpel formation. hec1 shows no alteration in fruit phenotype. hec3 transmitting tract was smaller in size in both the septum and the style, mutant carpels showed significantly fewer pollen tubes and pollination events, particularly in the basal half of the gynoecium.  AtbHLH040, INDEHISCENT (IND). |
| **Orphans** | AtbHLH108 |  | TcbHLH087 |  | AtbHLH108, maternal effect embryo arrest 8 (MEE8) |
