# Supplementary material for: The Basic/Helix-Loop-Helix Protein Family in Gossypium: Reference Genes and Their Evolution during Tetraploidization
Source: PLoS One. 2015 May 18;10(5):e0126558. doi: 10.1371/journal.pone.0126558 (PMC4436304; doi:10.1371/journal.pone.0126558)

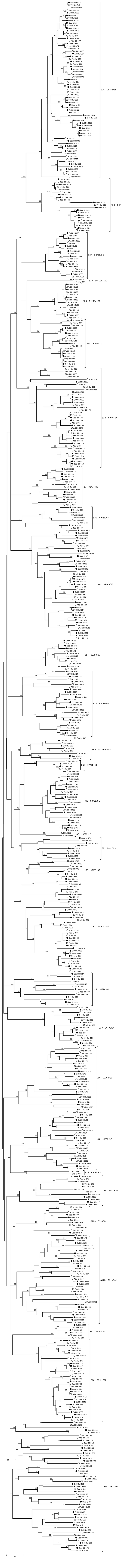

Supplement: S2 Fig — The phylogenetic trees were constructed and tested using NJ, ML, and MP methods on the basis of the alignment shown in S1 Fig. The NJ tree is presented, and subfamilies are indicated by square brackets with bootstraps (%) supported in NJ, ML and MP test (-, not supported). The scale bar indicates the estimated number of amino acid replacement per site. The sequences from A. thaliana (At), Gossypium (Go) and T. cacao (Tc) are marked with solid dots, open circles and squares, respectively. (PDF) [file pone.0126558.s002.pdf]
